# Multifaceted Roles for Macrophages in Prostate Cancer Skeletal Metastasis

**DOI:** 10.3389/fendo.2018.00247

**Published:** 2018-05-18

**Authors:** Chen Hao Lo, Conor C. Lynch

**Affiliations:** ^1^Cancer Biology Program, University of South Florida, Tampa, FL, United States; ^2^Tumor Biology Department, H. Lee Moffitt Cancer Center and Research Institute, Tampa, FL, United States

**Keywords:** bone, prostate cancer, metastasis, macrophage, polarization, therapy

## Abstract

Bone-metastatic prostate cancer is common in men with recurrent castrate-resistant disease. To date, therapeutic focus has largely revolved around androgen deprivation therapy (ADT) and chemotherapy. While second-generation ADTs and combination ADT/chemotherapy approaches have been successful in extending overall survival, the disease remains incurable. It is clear that molecular and cellular components of the cancer-bone microenvironment contribute to the disease progression and potentially to the emergence of therapy resistance. In bone, metastatic prostate cancer cells manipulate bone-forming osteoblasts and bone-resorbing osteoclasts to produce growth and survival factors. While osteoclast-targeted therapies such as bisphosphonates have improved quality of life, emerging data have defined important roles for additional cells of the bone microenvironment, including macrophages and T cells. Disappointingly, early clinical trials with checkpoint blockade inhibitors geared at promoting cytotoxic T cell response have not proved as promising for prostate cancer compared to other solid malignancies. Macrophages, including bone-resident osteomacs, are a major component of the bone marrow and play key roles in coordinating normal bone remodeling and injury repair. The role for anti-inflammatory macrophages in the progression of primary prostate cancer is well established yet relatively little is known about macrophages in the context of bone-metastatic prostate cancer. The focus of the current review is to summarize our knowledge of macrophage contribution to normal bone remodeling and prostate-to-bone metastasis, while also considering the impact of standard of care and targeted therapies on macrophage behavior in the tumor-bone microenvironment.

## Introduction

In 2018 alone, approximately 28,000 deaths from prostate cancer are predicted (1). While early stage disease is often treated successfully with surgery, radiation, and/or androgen deprivation therapy (ADT), advanced prostate cancer remains a moving target. Advanced disease typically manifests in the skeleton where metastases are often sensitive to first- and second-generation ADT. However, in a short period, the cancer becomes castrate resistant. In bone, prostate cancer causes extensive bone remodeling and formation that result in intense pain and heightened risk of pathologic fracture (2). These symptoms drastically reduce the patients’ quality of life and contribute substantially to disease morbidity and mortality. Bone-metastatic castrate-resistant prostate cancer (mCRPC) is currently incurable and appears to be refractory to recent advances in immunotherapy, such as checkpoint inhibitors (3–5). However, immune-based therapies such as Sipuleucel-T have been beneficial for some patients indicating that there may be room for alternative strategies in targeting the immune microenvironment of bone mCRPC. Despite macrophages constituting 8–15% of healthy adult male bone marrow, their role in the context of the bone-metastatic CRPC remains relatively underexplored.

## Macrophage Function in Tissue Homeostasis

Macrophages are phagocytic cells of the innate immune system responsible for maintenance of tissue homeostasis. Myeloid in nature and originating from hematopoietic stem cells that mature and differentiate into myeloblasts and monocytes, macrophages are noted for their diverse morphology and function across various tissues (6–8). For example, microglia are residential macrophages of the brain and play an important role in regulating synapse behavior (9). These cells have further demonstrated roles in immune modulation of inflammatory response to brain trauma at the blood–brain barrier (10). Other organ-specific macrophages include kupffer cells which turnover heme molecules through phagocytosis and degradation of hemoglobin in the liver (11, 12), and alveolar macrophages which engulf and eliminate dust particulates and microbes from the air on the luminal side of the mucosal epithelium lining in the lung (13, 14). Precursor and mature macrophages derived from the bone marrow also circulate the body, surveying and infiltrating sites of injury and infection to regulate local responses. Macrophages are known for their plasticity, and depending on signaling cues, can polarize into pro- or anti-inflammatory phenotypes. Traditionally, these phenotypes have been referred to as M1 and M2, but more recently it has been recognized that there are a spectrum of phenotypes across the M1/M2 continuum. Inflammatory stimuli released by necrotic or damaged tissue, such as interferon-gamma (IFNγ), interleukin-12 (IL-12), and reactive oxygen species (ROS) promote polarization into a pro-inflammatory phenotype (15–19), leading to the secretion of pro-apoptotic cytokines such as tumor necrosis factor (TNF) to induce apoptosis of neighboring cells. Pro-inflammatory macrophages can remove apoptotic neutrophils and cellular debris through phagocytosis and efferocytosis (20–24) and participate in the adaptive immune response by presenting disease-associated antigens to T and B cells that specifically target infectious agents or diseased cells (25–27). Following injury/infection resolution, secretion of factors including interleukin-10 (IL-10) and transforming growth factor beta (TGFβ) by fibroblasts and platelets promote the polarization of anti-inflammatory macrophages (28). Anti-inflammatory macrophages suppress further inflammation by secreting TGFβ, vascular endothelial growth factor (VEGF), and ROS that will deactivate T cells and promote T_H_2 response (29–32). These factors will also stimulate expansion of fibroblasts, endothelial cells, and other cell types for tissue repair (33, 34).

## Macrophage Roles in Bone Remodeling and Injury Repair

In the bone marrow, osteoclasts and osteoblasts are bone-specific cell populations that serve to resorb and mineralize the bone, respectively. The activities of these two populations are tightly coupled to ensure balanced bone turnover as well as returning the bone to homeostasis subsequent to injury. Osteoclasts are found residing on osteal surfaces and are histologically characterized as tartrate-resistant acid phosphatase (TRAP) positive and multi-nucleated (35, 36). Osteoclasts migrate to sites of active bone remodeling by chemotaxis, where they are involved in demineralization and resorption of the bone matrix (37–39). Upon apoptosis of the osteoclast, mesenchymal stem cell-derived osteoblasts rebuild the bone matrix *via* the deposition of type I collagen and hydroxyapatite (40). Traditionally, due to their myeloid origins and bone-specific functions, osteoclasts are considered the bone-resident macrophage population. However, roles for pro- and anti-inflammatory macrophages in controlling and coordinating osteoclast and osteoblast bone remodeling have been described. For example, IFNγ- and IL-12-stimulated NOS2 and TNF positive pro-inflammatory macrophages can promote osteoclast formation and bone resorption (41, 42). Conversely, anti-inflammatory macrophages are thought to contribute to bone formation (43).

A distinct population of bone-resident macrophages, osteomacs, has been described, and recent studies have shown important roles for these cells in modulating osteoblast activity in both bone homeostasis and injury repair (44). Osteomacs are morphologically characterized as mononuclear cells that form canopy-like structures around osteoblasts and can occupy as much as 75% of both murine and human endosteal and trabecular bone surfaces that are under active remodeling (45–48). Histologically, osteomacs are distinct from osteoclasts and are F4/80 positive but TRAP negative. Additionally, other groups have shown osteomacs to express common macrophage markers such as CD68, and also more specific markers, such as Mac-3 and CD169 (45, 46, 49). While osteomacs can be stimulated by receptor activator of nuclear kappa B ligand (RANKL) and colony stimulating factor-1 (CSF-1/M-CSF) to become osteoclasts *in vitro*, monocytes and other myeloid precursors were found to be more efficient osteoclast precursors (45). These data indicate that osteomacs are a plastic, yet distinct cell type, with specific functions in the bone marrow microenvironment. Indeed, further studies have revealed that osteomacs have diverse roles in regulating osteogenesis and osteolysis. Osteoblasts become inefficient as they age and need to be replenished to ensure proper homeostatic bone turnover (46). During normal bone turnover, osteomacs engulf apoptotic osteoblasts in a process called efferocytosis, which induces the secretion of TGFβ, TNF, and oncostatin M that facilitate osteoblastogenesis and bone formation (45, 46, 48). This mechanism has been confirmed in various *in vitro* and *in vivo* contexts. For example, removal of osteomacs from bone marrow-derived osteogenic co-cultures reduced osteoblast number and osteoblastic mineralization (47). The MAcrophage Fas-Induced Apoptosis (MAFIA) murine model is one in which administration of ligand AP20187 can systemically suppresses macrophage differentiation. Reduced osteoblast occupancy of the endosteal bone surfaces was observed in maturing MAFIA mice following AP20187 administration (47, 50). Congruently, parathyroid hormone-induced bone anabolism in the MAFIA model was suppressed upon macrophage ablation (51). Interestingly, when murine macrophages were depleted by clodronate liposome-induced apoptosis, osteoblast numbers remained stable (47, 50). Further comparison between two methods of macrophage depletion showed that transient macrophage apoptosis induced osteomac expansion and efferocytosis, which further enhanced osteoblast activity (46, 51, 52). Additionally, C57BL/6 mice bone marrow treated with trabectedin, a chemotherapy antagonist of macrophages, showed diminished phagocytic genetic signature, efferocytotic osteomac-induced RUNX2 positive osteoblastogenesis, and associated BV/TV status (53). During bone fracture repair, osteomacs can also sense apoptotic damaged cells and in response, initiate inflammation and immune recruitment through secretion of immune attractant factors, such as chemokine (C–C motif) ligand 2 (CCL2) and M-CSF (48). Additionally, LPS-stimulated osteomacs express TNF and NOS2, and suppress osteoblast activity *in vitro* (45). *In vivo*, bone fracture induced pro-inflammatory polarization of immune macrophages and osteomacs to secrete TNF and IFNβ, driving osteoclastogenesis and osteolysis (45). In fact, osteomacs have been shown to associate with osteoclasts at catabolic sites, substantiating their distinction from osteoclasts, and supporting their additional roles in regulating osteolysis (48). These studies indicate that osteomacs can direct the transition between osteolysis and osteogenesis by directly modulating the expansion and activity of osteoclasts and osteoblasts for repair in the event of bone injury (46). Taken together, these studies demonstrate the complex roles of bone-resident macrophages in bone remodeling (54, 55). How they contribute to the progression of bone-metastatic prostate cancer and respond to applied therapies has not been fully elucidated at this juncture.

## Macrophages Promote Primary Prostate Cancer Progression

Just as in other cancers, chronic inflammation in prostate cancer is thought to serve as a prelude to tumorigenesis (56). In fact, in cases of premalignant prostatic inflammatory atrophy, macrophages were observed coalescing at sites where inflammation-driven neoplasia caused disruptions in the epithelial lining of the prostate (57). In primary prostate cancer, pro- and anti-inflammatory tumor-associated macrophages (TAMs) have been found to comprise a significant portion of the immune cells infiltrating the tumor microenvironment with studies beginning to dissect roles for each population with regards to progression of the disease (58, 59). The exact pro- and anti-inflammatory constitution of TAMs vary across cancer types, but protective roles for TAMs have been described in prostate cancer. For example, macrophages located in the tumor-peripheral stroma correlated with increased recurrence-free survival (60), while macrophages expressing CD204, a marker associated with activation of antigen presentation in dendritic cells, correlate with better overall survival and prognosis (60–62). However, for the most part, macrophages have been found to contribute to, or directly promote, primary prostate cancer progression with individual patient cohort and meta-analysis studies identifying that macrophage infiltration correlates with disease aggressiveness and poor prognosis in prostate cancer (63–67). With respect to therapy, the density of anti-inflammatory macrophages in the primary disease correlates with extracapsular and biochemical recurrence following radical prostatectomy and/or ADT (63, 65, 66, 68).

The tumor-promoting roles of anti-inflammatory macrophages are thought to revolve around their immune-suppressive and angiogenic effects, both of which are important hallmarks of prostate cancer progression (68–71). Prostate cancer cells have been shown to secrete factors such as CSF-1 and CCL2 that lead to the recruitment of monocytes and macrophages that facilitate these processes (68, 72–78). Once recruited to the microenvironment, macrophages are exposed to a milieu of environmental cues that can drive their polarization into pro- or anti-inflammatory states (58). For example, exposure to tumor-derived IL-10 and -13 promotes macrophage polarization into an anti-inflammatory state. Subsequently, macrophages secrete factors, such as epidermal growth factor (EGF), platelet derived growth factors, and VEGF that promote cancer cell proliferation and angiogenesis of the tumor microenvironment (69, 79–83). Furthermore, ARG1 and TGFβ positive anti-inflammatory macrophages, along with myeloid-derived suppressor cells and regulatory T cells, collectively suppress inflammation and immune response within the tumor microenvironment (84–88). Both pro- and anti-inflammatory macrophages can also modulate T cell expansion and cytotoxicity by regulating the bioavailability of l-arginine, an important amino acid for T cell activity and survival (89). In addition, NOS2 positive pro-inflammatory macrophages synthesize nitric oxide that can promote T cell T_H_1 expansion (90, 91). Conversely, anti-inflammatory macrophages expand during T_H_2 response and additionally suppress T cell proliferation through expression of co-inhibitory molecule PD-L2 (30). Importantly, macrophages can also contribute to the activity of non-immune cells in the tumor microenvironment, such as cancer-associated fibroblasts (CAFs). Macrophage-secreted factors such as TGFβ are known potent regulators of CAFs that also promote tumor growth and invasion into the peripheral tissue to facilitate metastasis (68, 71, 92, 93).

## Macrophage Roles in Establishing the Pre-Metastatic Bone Marrow Niche?

While much is known about the role of macrophages in primary prostate cancer progression, less is known about how their polarization states in the bone marrow contribute to, or protect against prostate cancer metastasis to the bone and subsequent establishment. TNF, TGFβ, and VEGFA can be secreted by primary prostate cancer cells into circulation (94), which can activate marrow cell populations including bone-resident macrophages and hematopoietic progenitor cells. Furthermore, these tumor-derived factors have been shown to induce the recruitment of immunosuppressive myeloid populations into the bone that support immune evasion and ease the establishment of circulating tumor cells (95).

Emerging evidence has also defined important roles for prostate cancer-derived exosomes in the genesis of receptive pre-metastatic niches (96, 97). Exosomes are nanometer-sized vesicles that can be shed in large numbers by cancer cells. The cargo contents of cancer cell-derived exosomes vary greatly, but can contain cell-adhesion molecules, receptor tyrosine kinases, proteases, miRNAs and miRNA processing machinery, mRNA, and DNA (98, 99). Injection of mice with exosomes derived from human prostate cancer peripheral blood or murine prostate cancer cells lines (TRAMPc1) demonstrated impaired murine osteoclast formation and enhanced osteoblast differentiation suggesting that prostate cancer-derived exosomes play a role in tipping the balance toward bone formation, a common hallmark of bone-metastatic prostate cancer (100, 101). Milk fat globule-EGF factor 8 protein (MFG-E8) was found in human prostate cancer patient exosomes, and tissue biopsies. MFG-E8 has been shown to mediate macrophage efferocytosis of apoptotic osteoblasts and cancer cells; these macrophages then exhibit an anti-inflammatory phenotype and in turn promote immune suppression through expression of TGFβ and ARG1 (102, 103). Characterization of prostate cancer-derived exosomes has identified various proteins and miRNA that can promote metastasis. Among the miRNA identified, miRNA-21 is particularly interesting given that it is upregulated in bone-metastatic prostate cancer and has known roles in regulating osteoclasto- and osteoblastogenesis (23, 97, 104, 105). Additionally, miRNA-21 is known to regulate macrophage phagocytosis of necrotic or diseased tissue in the context of wounding (23). Other miRNA identified in prostate cancer-derived exosomes that can influence osteoclast and osteoblast differentiation include miRNA-128 and -183 (95, 97).

Collectively, these studies show that bone marrow macrophages contribute to bone-metastatic outgrowth of disseminated prostate cancers, whereby cancer-derived signals or exosomes significantly influence macrophage activity in the pre-metastatic niche. In turn, these changes appear to be permissive for prostate cancer cell colonization of bone.

## TAMs in Metastatic Cascade of Prostate Cancer

The role of TAM in the metastatic dissemination of primary prostate cancer has been extensively studied and reviewed. Here, we reference seminal review articles that outline the molecular and cellular communication between TAMs and primary prostate cancers resulting in tumor vascularization, epithelial-to-mesenchymal transition, intravasation, and eventual colonization of distal sites, including, specifically, the skeletal bone marrow (58, 83, 106–109).

## Macrophages in the Progression of Established Prostate to Bone Metastases

**Figure 1:**
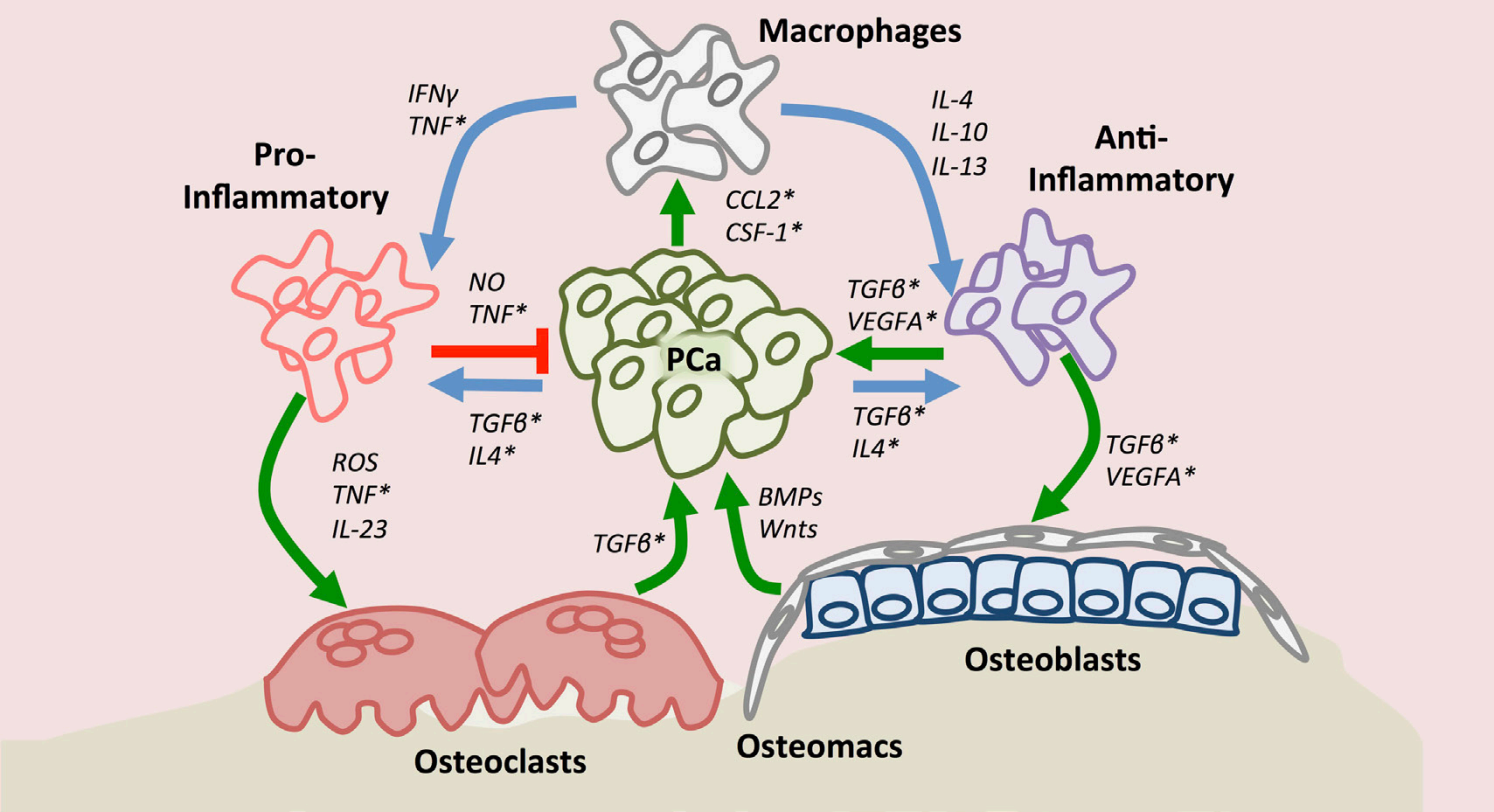
Macrophage roles in the context of the bone-metastatic prostate cancer. Upon recruitment to the site of metastasis by chemokine (C–C motif) ligand 2 and or colony stimulating factor-1, macrophages may polarize (blue arrows) into pro- or anti-inflammatory states depending on environmental cues. Tumor-associated macrophages have protective (red arrow) or contributory effects (green arrow) directly on prostate cancer. Importantly, macrophages, including bone-resident osteomacs, impact osteoclast and osteoblast function (green arrows) thereby also indirectly regulating prostate cancer progression in bone. Asterisks denote factors to which small molecule or biological inhibitors have been developed.

Once actively growing in the skeleton, prostate cancer cells manipulate the cells of the bone microenvironment to promote areas of extensive osteolysis and osteogenesis. Osteoclasts have traditionally been regarded as a specialized bone-resident macrophage population due to their myeloid lineage and phagocytic nature in bone resorption, which leads to the release of bone matrix-sequestered factors that feed the metastatic prostate cancer cells (110–112). While macrophages can fuse and form into osteoclasts in response to RANKL (113, 114), the role of individual macrophage populations in controlling prostate cancer bone interaction remains relatively underexplored. Recent observations in patient biopsies have implicated the role of osteal macrophages in established bone-metastatic prostate cancer (115). CD68 positive macrophages were detectable at high density within the tumor, whereas osteoclasts and osteomacs were found at the tumor-bone interface, suggesting potentially differential functions for each population in the growing lesions (115). Studies have also defined causal roles for macrophage populations in the growth of prostate cancer in bone. For example, intratibial inoculation of RM1 prostate cancer cells into macrophage-depleted bone marrow of MAFIA mice resulted in decreased pathologic osteolysis (107, 116). Additionally, depleting macrophages using clodronate liposome prior to tumor inoculation significantly limited cancer growth in bone (116). Further evidence supporting contributory roles for macrophages in the progression of bone-metastatic prostate cancer lesions has been provided using similar total macrophage depletion approaches (107, 115). Additionally, roles for osteomacs in the cancer-bone microenvironment have also been described, where CD169 positive tumor-associated osteomacs were found to facilitate tumor-induced pathologic osteogenesis. Interestingly, CD169 negative macrophages have been shown to promote tumor growth (115) and phenotypically resemble CD206 positive anti-inflammatory macrophages found in primary prostate cancer (109, 117). Taken together, these studies suggest that macrophages contribute to prostate cancer metastasis and growth in the bone microenvironment (Figure 1). However, deeper investigations into the precise roles of pro- and anti-inflammatory macrophages and osteomacs in the process are warranted.

## Macrophage Response to Standard of Care Treatments/Therapies

As discussed, macrophage polarization can have protective or contributory roles; however, the impact of standard of care approaches on macrophage behavior has not been explored in depth thus far. For men with bone-metastatic CRPC, treatment options largely focus on radiation therapy to alleviate pain and reduce tumor burden, or therapeutics that target the cancer cells, such as chemotherapy and ADT. Although castrate-resistant, CRPC prostate cancer cells remain dependent on androgen signaling *via* the expression of constitutively active androgen receptor splice variants, and/or autocrine expression of their own androgen (118–120). Underscoring this dependency on androgens or the AR receptor for survival, second-generation ADTs (enzalutamide and abiraterone) have been shown to significantly improve overall survival. In murine xenograft models, enzalutamide treatment of C4-2B and TRAMPc1 prostate tumors induced STAT3-mediated CCL2 expression and recruitment of CCR2 positive macrophages, enhancing angiogenesis and tumor invasion (121–123). Other second-generation ADTs, such as abiraterone, have also been shown to upregulate cancer cell CSF1 expression to promote macrophage infiltration, wound healing and, subsequently, tumor proliferation (75). Additionally, ADT drives tumor secretion of IL-10 and -13 that contribute to the polarization of macrophages into an anti-inflammatory phenotype (75). While in-depth studies have not examined the precise effects of second-generation ADT on macrophage behavior in bone-metastatic disease, it is plausible that the drugs may have actions similar to those noted at the primary site by promoting an anti-inflammatory phenotype. Critically, little work has been done to explore the role of ADT on bone-resident macrophages. As discussed, osteomacs appear to be key regulators of bone formation, and androgen depletion may impact the ability of osteomacs and osteoblasts to generate bone. This would be beneficial in reducing the aberrant osteogenesis associated with bone-metastatic prostate cancer, although it could promote systemic osteoporosis, a phenomenon noted in men undergoing chronic ADT treatment (124).

Taxane chemotherapies such as docetaxel and cabazitaxel are also used for the treatment of advanced prostate cancer patients. These drugs inhibit microtubule disassembly during mitotic chromosome segregation and induce apoptosis in neoplastic cells, and they are commonly given to patients with mCRPC who have failed ADT (125–128). Interestingly, for chemotherapy-sensitive CRPC, docetaxel has immune-stimulatory effects and can inhibit myeloid-derived suppressor cells, while promoting a switch in macrophages from an anti- to pro-inflammatory phenotype (129, 130). However, bone-metastatic CRPCs eventually become resistant to docetaxel, at which point they progress. In the case of chemotherapy-resistant cancer, the cancer cells can now secrete inflammatory cytokines such as IL-6 and -8, to recruit and differentiate monocytes and endothelial cells, for immune suppression and angiogenesis, respectively (131–134). Specifically, IL-6-induced mature macrophages are subsequently driven by other secreted cytokines such as IL-4 to anti-inflammatory states to induce immune suppression (131). IL-6 also induces prostate cancer survival by inducing Bcl/Stat-mediated survival signaling (131). Docetaxel can also induce CCL2 expression in cancer cells, a potent factor that not only induces prostate cancer growth and is correlated with disease progression but also recruits anti-inflammatory macrophages that drive tumor progression (74, 131, 135–139). Anti-inflammatory macrophages may also promote bone formation, but studies have shown that docetaxel impacts bone remodeling by suppressing osteoclast formation and osteoblast expansion, therefore, potentially off-setting the contribution of anti-inflammatory macrophages to cancer-induced bone disease (140).

Newer therapies being employed in the clinic may also have important effects on macrophage behavior in bone. For example, radium-223 is an alpha-emitting radionuclide that binds to calcium and promotes prostate cancer cell death in the neighboring vicinity. The treatment has been successful in extending the overall survival of men with bone-metastatic CRPC. The apoptosis induced by radium-223 may increase the bioavailability of tumor antigen in a cytotoxic microenvironment. Since macrophages are strong antigen presenting cells that mediate T cell antigenicity, it will be interesting to explore whether peripheral macrophages become pro-inflammatory and immune-stimulatory (141). However, the effects of radiation therapy can be double edged. In humans, myelosuppression, leucopenia, and lymphopenia, were noted in radium-223 patients (142, 143). Further, in multiple cancer models, radiotherapy has been demonstrated to enhance macrophage infiltration, and over time, the polarization of macrophages into an anti-inflammatory phenotype may promote angiogenesis and cancer cell survival/recurrence (144–146). Taken together, these studies indicate that while applied therapies are initially successful in limiting disease progression, the emergence of resistant disease is often coupled/correlated with changes in macrophage polarization. Whether chronic exposure to standard of care therapies alters the microenvironment which in turn facilitates the emergence of resistant cancer cells remains to be determined. Conversely, little is known as to whether the evolution of resistant cancer cells in response to therapy impacts the behavior of the surrounding microenvironment.

## Can Macrophage-Based Therapies be Impactful for the Treatment of Bone-Metastatic Prostate Cancer?

The addition of therapies geared at blocking macrophage function, in particular anti-inflammatory function, in combination with standard of care treatments may yield more effective and durable responses in addition to preventing the recurrence of resistant disease. The role of macrophages in promoting the progression of numerous solid malignancies has been described, and as a consequence, translational studies have been geared toward the development of targeted therapies that can either deplete myeloid populations and/or alter the polarization status of macrophages. Numerous factors control macrophage infiltration and polarization but can have dual tumor-promoting and -protective effects. For example, correlative and causal roles for TNF in the progression and metastasis of prostate cancer have been described (147–153). Given the roles of TNF in inflammatory diseases, such as arthritis, it is unsurprising that biologicals targeting either the ligand or the receptor have been an active area of research. In the cancer setting, TNF can promote tumor growth and angiogenesis with preclinical trials demonstrating efficacy for TNF blocking reagents (154). Conversely, however, tumor-protective roles have been described in addition to potential risks for the development of cancers such as soft tissue sarcoma. This, combined with the potential for adverse toxicity associated with TNF inhibition, has diminished enthusiasm for the application of TNF inhibitors in the cancer setting. However, more encouraging results for other targets that impact macrophage behavior have been noted including, CCL-2/CCR-2, IL-4, and CSF1 receptor (CSF1R).

### CCL-2

Chemokine (C–C motif) ligand 2 is expressed by prostate cancer cells, and while it can promote cell growth and invasion in an autocrine manner, it has also been shown to be a key driver of CCR2-expressing (CCL2 receptor) macrophages and monocyte recruitment (73, 155, 156). Moreover, the role of CCL2 seems particularly relevant in the context of bone-metastatic disease, where CCL2-expressing prostate cancers recruit endothelial cells and osteoblasts to drive angiogenesis and osteogenesis, respectively, both of which enhance the progression of the disease (73, 157). Underscoring the importance of CCL2 in the tumor-bone microenvironment, studies demonstrated that neutralization of CCL2 with a monoclonal antibody (C1142) was successful in both attenuating tumor growth as well as bone pathology in various preclinical models (156, 158). As a result, the humanized version, CNTO 888 (Carlumab), was developed to neutralize CCL2 signaling function in advanced prostate cancer. While the drug was well-tolerated in clinical trials, no anti-tumor activity was noted as a single agent for the treatment of metastatic CRPC (158–161). Given that targeting CCL2, or the receptor CCR2, in other diseases has been shown to be impactful in reducing inflammatory responses, it is possible that combination with standard of care treatments may result in more profound effects. Interestingly, heightened levels of CCL2 were noted in patients that developed resistance to docetaxel, and pre-clinical studies in which docetaxel and C1142 were combined demonstrated significant inhibition of bone-metastatic cancer growth and associated bone disease (139, 162, 163). Surprisingly, a phase I clinical trial combining docetaxel with CNTO 888 demonstrated tolerability but not a suppression of serum CCL2 levels or tumor response. This may indicate that higher dosing is required to block the CCL2–CCR2 axis or a combination of CNTO 888 with CCR2-specific antibodies such as MLN1202 is needed to achieve effective responses in humans (164). In addition to potentially depleting macrophages from the bone-tumor microenvironment, CCL2/CCR2 therapies can also reduce osteoclast recruitment and formation thereby protecting the patient from skeletal-related events such as pathologic fracture (157, 165).

### Interleukin 4 (IL-4)/IL-4R

Interleukin 4 is an anti-inflammatory cytokine found upregulated in various solid malignancies that can promote tumor growth by driving anti-inflammatory macrophage polarization which in turn facilitates tumor proliferation, angiogenesis, and metastasis (166–168). This effect may be concentration dependent as high levels of IL-4 have an anti-tumor effect (169–171). In prostate cancer, IL-4 trends with PSA expression and can stimulate IL-4 receptor (IL-4R) positive prostate cancer cells to grow and metastasize *via* downstream activation of JAK/STAT6 pathway (172). IL-4 can also promote anti-tumor immunity. While IL-4 supports proliferation of T cells, it converts mature CD8 T cells from T_H_1 to T_H_2 response; this transition suppresses their cytolytic potential and leads to immune evasion and tolerance (168). IL-4 expression is especially heightened in hormone-refractory versus hormone-sensitive prostate cancer (169, 172). In the context of ADT, studies have shown that IL-4 can induce AR signaling reactivation, independent of androgen, suggesting IL-4 over expression as a resistance mechanism to restore cancer growth in androgen-depleted prostate cancer (172, 173). Combination of anti-IL-4 agents with ADT may, therefore, extend tumor ADT sensitivity. To this end, IL-4-targeted therapies are in development for the treatment of asthma and allergic responses. However, the anti-cancer effects of the therapy could be lessened due to the potential impact of IL-4 blockade on the activity of cytotoxic immune cells. Adverse systemic effects may also be an issue, but strategies that focus on cancer cell- or TAM-specific delivery may be of use. Furthermore, IL-4 has been shown to limit osteoblast proliferation and induce the expression of IL-6 while inhibiting osteoclastogenesis (174, 175). Therefore, while inhibiting IL-4 may exacerbate the cancer-associated bone disease, it would also inhibit IL-6 expression by osteoblasts which in turn may prevent macrophage-mediated resistance to chemotherapy (176).

### CSF1/CSF1R

Prostate cancer-derived colony stimulatory factor 1 (CSF1) can lead to the recruitment of CSF1R positive macrophages. In the tumor, CSF1 signaling promotes macrophage survival and polarization into an ARG1, CD206, and IL-10 positive anti-inflammatory phenotype, while simultaneously inhibiting a NOS2 and IL-12 positive pro-inflammatory phenotype (75–77). Additionally, tumor-derived CSF1 recruits MDSC, and these immunosuppressive myeloid infiltrates are particularly important in tumor survival and progression (76). Interestingly, standard of care therapies such as radiation and ADT promote CSF1 expression by prostate cancer cells leading to increased infiltration of macrophages (75, 77). The CSF1/CSF1R axis is known to play a role in macrophage infiltration and anti-inflammatory polarization in other cancers and several anti-CSF1R agents have been developed, including GW2580 and PLX3397. These agents have demonstrated significant success in abrogating therapy-induced CSF1R positive macrophage infiltration using animal models of cancer progression, including prostate cancer (75, 77, 177–179). Further, treatment with ADT and PLX3397 or GW2580 reduced macrophage infiltration compared to either therapy as a single agent (75). This indicates that combination of ADT and anti-CSF1R therapy would be clinically beneficial. Currently, several clinical trials are ongoing that will test the efficacy and impact of CSF/CSF1R inhibitors. For prostate cancer, recent studies have shown that PLX3397 delays the emergence of CRPC by reducing the number of infiltrating TAM, and a phase II clinical trial was performed in a small cohort of bone-metastatic CRPC patients with results pending (NCT 01499043). Various other combination therapy studies for prostate cancer using ADT with PLX3397 and other anti-CSF1R agents are underway and it will be interesting to see how well they perform relative to when used as single agents (78). Of note, blockade of CSF1R signaling in mice significantly reduced osteoclast number, leading to increased bone mass that may be useful in offsetting ADT-associated osteoporosis (180).

## Conclusion

Bone-metastatic CRPC is currently incurable and will be present in over 90% of the men who succumb to the disease. While ADTs and chemotherapy have improved overall survival rates, more work is required to help in controlling and/or eradicating the disease. This can be achieved by understanding the cellular and molecular mechanisms involved. To this end, clear roles for the stromal and immune components of the tumor microenvironment have been described. Macrophages represent a large component of the immune infiltrate, and depending on their polarization state, can contribute to the progression of the disease. Many standard of care therapies focus on elimination of the cancer cell but indirectly, these therapies also impact the behavior of the surrounding macrophage population and lessen therapeutic efficacy. The factors controlling macrophage infiltration and polarization are the focus of translational efforts with several reagents in clinical trials. Combination therapies such as ADT with anti-CCL2/CCR2 or anti-CSF1R inhibitors may prove to significantly extend the overall survival of men with bone-metastatic CRPC. Further, given the role of macrophages in controlling bone remodeling, dampening macrophage activity may reduce prostate cancer-induced osteogenesis, thereby directly improving patient quality of life.

## Author Contributions

CHL and CCL wrote and edited the review.

## Conflict of Interest Statement

The authors declare that the research was conducted in the absence of any commercial or financial relationships that could be construed as a potential conflict of interest.

## References

[1] American Cancer Society. Cancer Facts and Figures. (2018). Available from: https://www.cancer.org/research/cancer-facts-statistics.html (Accessed: March 1, 2018).

[2] Roudier MP True LD Higano CS Vesselle H Ellis W Lange P Phenotypic heterogeneity of end-stage prostate carcinoma metastatic to bone. Hum Pathol (2003) 34:646–53. 10.1016/S0046-8177(03)00190-4 12874759

[3] Modena A Ciccarese C Iacovelli R Brunelli M Montironi R Fiorentino M Immune checkpoint inhibitors and prostate cancer: a new frontier? Oncol Rev (2016) 10:293. 10.4081/oncol.2016.293 27471580 PMC4943092

[4] Goswami S Aparicio A Subudhi SK Immune checkpoint therapies in prostate cancer. Cancer J (2016) 22:117–20. 10.1097/PPO.0000000000000176 27111907 PMC4847149

[5] Reinstein ZZ Pamarthy S Sagar V Costa R Abdulkadir SA Giles FJ Overcoming immunosuppression in bone metastases. Crit Rev Oncol Hematol (2017) 117:114–27. 10.1016/j.critrevonc.2017.05.004 28600175

[6] Alliot F Godin I Pessac B. Microglia derive from progenitors, originating from the yolk sac, and which proliferate in the brain. Brain Res Dev Brain Res (1999) 117:145–52. 10.1016/S0165-3806(99)00113-3 10567732

[7] Gomez Perdiguero E Klapproth K Schulz C Busch K Azzoni E Crozet L Tissue-resident macrophages originate from yolk-sac-derived erythro-myeloid progenitors. Nature (2015) 518:547–51. 10.1038/nature13989 25470051 PMC5997177

[8] van de Laar L Saelens W De Prijck S Martens L Scott CL Van Isterdael G Yolk Sac macrophages, fetal liver, and adult monocytes can colonize an empty niche and develop into functional tissue-resident macrophages. Immunity (2016) 44:755–68. 10.1016/j.immuni.2016.02.017 26992565

[9] Thomas WE. Brain macrophages: evaluation of microglia and their functions. Brain Res Brain Res Rev (1992) 17:61–74. 10.1016/0165-0173(92)90007-9 1638276

[10] Gehrmann J Matsumoto Y Kreutzberg GW. Microglia: intrinsic immuneffector cell of the brain. Brain Res Brain Res Rev (1995) 20:269–87. 10.1016/0165-0173(94)00015-H 7550361

[11] Haubrich WS Kupffer of Kupffer cells. Gastroenterology (2004) 127:16 10.1053/j.gastro.2004.05.041 15236167

[12] Naito M Hasegawa G Takahashi K. Development, differentiation, and maturation of Kupffer cells. Microsc Res Tech (1997) 39:350–64. 10.1002/(SICI)1097-0029(19971115)39:4<350::AID-JEMT5>3.0.CO;2-L 9407545

[13] Hussell T Bell TJ. Alveolar macrophages: plasticity in a tissue-specific context. Nat Rev Immunol (2014) 14:81–93. 10.1038/nri3600 24445666

[14] Joshi N Walter JM Misharin AV. Alveolar macrophages. Cell Immunol (2018) pii: S0008-8749(18)30005–4. 10.1016/j.cellimm.2018.01.005 29370889

[15] Tan HY Wang N Li S Hong M Wang X Feng Y. The reactive oxygen species in macrophage polarization: reflecting its dual role in progression and treatment of human diseases. Oxid Med Cell Longev (2016) 2016:2795090. 10.1155/2016/2795090 27143992 PMC4837277

[16] Liu YC Zou XB Chai YF Yao YM. Macrophage polarization in inflammatory diseases. Int J Biol Sci (2014) 10:520–9. 10.7150/ijbs.8879 24910531 PMC4046879

[17] Zhou D Huang C Lin Z Zhan S Kong L Fang C Macrophage polarization and function with emphasis on the evolving roles of coordinated regulation of cellular signaling pathways. Cell Signal (2014) 26:192–7. 10.1016/j.cellsig.2013.11.004 24219909

[18] Sica A Mantovani A. Macrophage plasticity and polarization: in vivo veritas. J Clin Invest (2012) 122:787–95. 10.1172/JCI59643 22378047 PMC3287223

[19] Wang N Liang H Zen K Molecular mechanisms that influence the macrophage M1–M2 polarization balance. Front Immunol (2014) 5:614 10.3389/fimmu.2014.00614 25506346 PMC4246889

[20] Allen JE Ruckerl D. The silent undertakers: macrophages programmed for efferocytosis. Immunity (2017) 47:810–2. 10.1016/j.immuni.2017.10.010 29166582

[21] Chang CF Goods BA Askenase MH Hammond MD Renfroe SC Steinschneider AF Erythrocyte efferocytosis modulates macrophages towards recovery after intracerebral hemorrhage. J Clin Invest (2018) 128:607–24. 10.1172/JCI95612 29251628 PMC5785262

[22] Campana L Starkey Lewis PJ Pellicoro A Aucott RL Man J O’Duibhir E The STAT3-IL-10-IL-6 pathway is a novel regulator of macrophage efferocytosis and phenotypic conversion in sterile liver injury. J Immunol (2018) 200:1169–87. 10.4049/jimmunol.1701247 29263216 PMC5784823

[23] Das A Ganesh K Khanna S Sen CK Roy S. Engulfment of apoptotic cells by macrophages: a role of microRNA-21 in the resolution of wound inflammation. J Immunol (2014) 192:1120–9. 10.4049/jimmunol.1300613 24391209 PMC4358325

[24] Michlewska S Dransfield I Megson IL Rossi AG. Macrophage phagocytosis of apoptotic neutrophils is critically regulated by the opposing actions of pro-inflammatory and anti-inflammatory agents: key role for TNF-alpha. FASEB J (2009) 23:844–54. 10.1096/fj.08-121228 18971259

[25] Roberts CA Dickinson AK Taams LS. The interplay between monocytes/macrophages and CD4(+) T cell subsets in rheumatoid arthritis. Front Immunol (2015) 6:571. 10.3389/fimmu.2015.00571 26635790 PMC4652039

[26] Pozzi LA Maciaszek JW Rock KL. Both dendritic cells and macrophages can stimulate naive CD8 T cells in vivo to proliferate, develop effector function, and differentiate into memory cells. J Immunol (2005) 175:2071–81. 10.4049/jimmunol.175.4.2071 16081773

[27] Arango Duque G Descoteaux A Macrophage cytokines: involvement in immunity and infectious diseases. Front Immunol (2014) 5:491 10.3389/fimmu.2014.00491 25339958 PMC4188125

[28] Pakyari M Farrokhi A Maharlooei MK Ghahary A. Critical role of transforming growth factor beta in different phases of wound healing. Adv Wound Care (New Rochelle) (2013) 2:215–24. 10.1089/wound.2012.0406 24527344 PMC3857353

[29] Kraaij MD Savage ND van der Kooij SW Koekkoek K Wang J van den Berg JM Induction of regulatory T cells by macrophages is dependent on production of reactive oxygen species. Proc Natl Acad Sci U S A (2010) 107:17686–91. 10.1073/pnas.1012016107 20861446 PMC2955141

[30] Huber S Hoffmann R Muskens F Voehringer D. Alternatively activated macrophages inhibit T-cell proliferation by Stat6-dependent expression of PD-L2. Blood (2010) 116:3311–20. 10.1182/blood-2010-02-271981 20625006

[31] Schebesch C Kodelja V Muller C Hakij N Bisson S Orfanos CE Alternatively activated macrophages actively inhibit proliferation of peripheral blood lymphocytes and CD4+ T cells in vitro. Immunology (1997) 92:478–86. 10.1046/j.1365-2567.1997.00371.x 9497489 PMC1364153

[32] Gelderman KA Hultqvist M Pizzolla A Zhao M Nandakumar KS Mattsson R Macrophages suppress T cell responses and arthritis development in mice by producing reactive oxygen species. J Clin Invest (2007) 117:3020–8. 10.1172/JCI31935 17909630 PMC1994618

[33] Minutti CM Knipper JA Allen JE Zaiss DM. Tissue-specific contribution of macrophages to wound healing. Semin Cell Dev Biol (2017) 61:3–11. 10.1016/j.semcdb.2016.08.006 27521521

[34] Mantovani A Biswas SK Galdiero MR Sica A Locati M. Macrophage plasticity and polarization in tissue repair and remodelling. J Pathol (2013) 229:176–85. 10.1002/path.4133 23096265

[35] Yavropoulou MP Yovos JG Osteoclastogenesis – current knowledge and future perspectives. J Musculoskelet Neuronal Interact (2008) 8:204–16. 18799853

[36] Asagiri M Takayanagi H. The molecular understanding of osteoclast differentiation. Bone (2007) 40:251–64. 10.1016/j.bone.2006.09.023 17098490

[37] Teitelbaum SL. Bone resorption by osteoclasts. Science (2000) 289:1504–8. 10.1126/science.289.5484.1504 10968780

[38] Nijweide PJ Burger EH Feyen JH Cells of bone: proliferation, differentiation, and hormonal regulation. Physiol Rev (1986) 66:855–86. 10.1152/physrev.1986.66.4.855 3532144

[39] Schindeler A McDonald MM Bokko P Little DG. Bone remodeling during fracture repair: the cellular picture. Semin Cell Dev Biol (2008) 19:459–66. 10.1016/j.semcdb.2008.08.008 18692584

[40] McArdle A Marecic O Tevlin R Walmsley GG Chan CK Longaker MT The role and regulation of osteoclasts in normal bone homeostasis and in response to injury. Plast Reconstr Surg (2015) 135:808–16. 10.1097/PRS.0000000000000963 25719699

[41] Yamaguchi T Movila A Kataoka S Wisitrasameewong W Ruiz Torruella M Murakoshi M Proinflammatory M1 macrophages inhibit RANKL-induced osteoclastogenesis. Infect Immun (2016) 84:2802–12. 10.1128/IAI.00461-16 27456834 PMC5038061

[42] Huang R Wang X Zhou Y Xiao Y. RANKL-induced M1 macrophages are involved in bone formation. Bone Res (2017) 5:17019. 10.1038/boneres.2017.19 29263936 PMC5645773

[43] Sesia SB Duhr R Medeiros da Cunha C Todorov A Schaeren S Padovan E Anti-inflammatory/tissue repair macrophages enhance the cartilage-forming capacity of human bone marrow-derived mesenchymal stromal cells. J Cell Physiol (2015) 230:1258–69. 10.1002/jcp.24861 25413299

[44] Hume DA Loutit JF Gordon S. The mononuclear phagocyte system of the mouse defined by immunohistochemical localization of antigen F4/80: macrophages of bone and associated connective tissue. J Cell Sci (1984) 66:189–94. 6378941 10.1242/jcs.66.1.189

[45] Pettit AR Chang MK Hume DA Raggatt L-J. Osteal macrophages: a new twist on coupling during bone dynamics. Bone (2008) 43:976–82. 10.1016/j.bone.2008.08.128 18835590

[46] Sinder BP Pettit AR McCauley LK. Macrophages: their emerging roles in bone. J Bone Miner Res (2015) 30:2140–9. 10.1002/jbmr.2735 26531055 PMC4876707

[47] Chang MK Raggatt LJ Alexander KA Kuliwaba JS Fazzalari NL Schroder K Osteal tissue macrophages are intercalated throughout human and mouse bone lining tissues and regulate osteoblast function in vitro and in vivo. J Immunol (2008) 181:1232–44. 10.4049/jimmunol.181.2.1232 18606677

[48] Batoon L Millard SM Raggatt LJ Pettit AR. Osteomacs and bone regeneration. Curr Osteoporos Rep (2017) 15:385–95. 10.1007/s11914-017-0384-x 28647885

[49] Batoon L Millard SM Wullschleger ME Preda C Wu AC Kaur S CD169(+) macrophages are critical for osteoblast maintenance and promote intramembranous and endochondral ossification during bone repair. Biomaterials (2017). 10.1016/j.biomaterials.2017.10.033 29107337

[50] Alexander KA Raggatt L-J Millard S Batoon L Chiu-Ku Wu A Chang M-K Resting and injury-induced inflamed periosteum contain multiple macrophage subsets that are located at sites of bone growth and regeneration. Immunol Cell Biol (2016) 95:7–16. 10.1038/icb.2016.74 27553584

[51] Cho SW Soki FN Koh AJ Eber MR Entezami P Park SI Osteal macrophages support physiologic skeletal remodeling and anabolic actions of parathyroid hormone in bone. Proc Natl Acad Sci U S A (2014) 111:1545–50. 10.1073/pnas.1315153111 24406853 PMC3910564

[52] Shi M Wang C Wang Y Tang C Miron RJ Zhang Y. Deproteinized bovine bone matrix induces osteoblast differentiation via macrophage polarization. J Biomed Mater Res A (2017) 106:1236–46. 10.1002/jbm.a.36321 29280261

[53] Sinder BP Zweifler L Koh AJ Michalski MN Hofbauer LC Aguirre JI Bone mass is compromised by the chemotherapeutic trabectedin in association with effects on osteoblasts and macrophage efferocytosis. J Bone Miner Res (2017) 32:2116–27. 10.1002/jbmr.3196 28600866 PMC5640484

[54] Bozec A Soulat D. Latest perspectives on macrophages in bone homeostasis. Pflügers Arch (2017) 469:517–25. 10.1007/s00424-017-1952-8 28247013

[55] Kaur S Raggatt LJ Batoon L Hume DA Levesque JP Pettit AR. Role of bone marrow macrophages in controlling homeostasis and repair in bone and bone marrow niches. Semin Cell Dev Biol (2017) 61:12–21. 10.1016/j.semcdb.2016.08.009 27521519

[56] Sfanos KS De Marzo AM. Prostate cancer and inflammation: the evidence. Histopathology (2012) 60:199–215. 10.1111/j.1365-2559.2011.04033.x 22212087 PMC4029103

[57] De Marzo AM Marchi VL Epstein JI Nelson WG. Proliferative inflammatory atrophy of the prostate: implications for prostatic carcinogenesis. Am J Pathol (1999) 155:1985–92. 10.1016/S0002-9440(10)65517-4 10595928 PMC1866955

[58] Pollard JW Tumour-educated macrophages promote tumour progression and metastasis. Nat Rev Cancer (2004) 4:71–8. 10.1038/nrc1256 14708027

[59] Comito G Giannoni E Segura CP Barcellos-de-Souza P Raspollini MR Baroni G Cancer-associated fibroblasts and M2-polarized macrophages synergize during prostate carcinoma progression. Oncogene (2014) 33:2423–31. 10.1038/onc.2013.191 23728338

[60] Takayama H Nonomura N Nishimura K Oka D Shiba M Nakai Y Decreased immunostaining for macrophage scavenger receptor is associated with poor prognosis of prostate cancer. BJU Int (2009) 103:470–4. 10.1111/j.1464-410X.2008.08013.x 18778349

[61] Shimura S Yang G Ebara S Wheeler TM Frolov A Thompson TC. Reduced infiltration of tumor-associated macrophages in human prostate cancer: association with cancer progression. Cancer Res (2000) 60:5857–61. 11059783

[62] Cao J Liu J Xu R Zhu X Zhao X Qian BZ. Prognostic role of tumour-associated macrophages and macrophage scavenger receptor 1 in prostate cancer: a systematic review and meta-analysis. Oncotarget (2017) 8:83261–9. 10.18632/oncotarget.18743 29137340 PMC5669966

[63] Lissbrant IF Stattin P Wikstrom P Damber JE Egevad L Bergh A. Tumor associated macrophages in human prostate cancer: relation to clinicopathological variables and survival. Int J Oncol (2000) 17:445–51. 10.3892/ijo.17.3.445 10938382

[64] Bingle L Brown NJ Lewis CE. The role of tumour-associated macrophages in tumour progression: implications for new anticancer therapies. J Pathol (2002) 196:254–65. 10.1002/path.1027 11857487

[65] Nonomura N Takayama H Nakayama M Nakai Y Kawashima A Mukai M Infiltration of tumour-associated macrophages in prostate biopsy specimens is predictive of disease progression after hormonal therapy for prostate cancer. BJU Int (2011) 107:1918–22. 10.1111/j.1464-410X.2010.09804.x 21044246

[66] Gollapudi K Galet C Grogan T Zhang H Said JW Huang J Association between tumor-associated macrophage infiltration, high grade prostate cancer, and biochemical recurrence after radical prostatectomy. Am J Cancer Res (2013) 3:523–9. 24224130 PMC3816972

[67] Lin D Wang X Choi SYC Ci X Dong X Wang Y. Immune phenotypes of prostate cancer cells: evidence of epithelial immune cell-like transition? Asian J Urol (2016) 3:195–202. 10.1016/j.ajur.2016.08.002 29264187 PMC5730833

[68] Lanciotti M Masieri L Raspollini MR Minervini A Mari A Comito G The role of M1 and M2 macrophages in prostate cancer in relation to extracapsular tumor extension and biochemical recurrence after radical prostatectomy. Biomed Res Int (2014) 2014:6. 10.1155/2014/486798 24738060 PMC3967497

[69] Hillen F Griffioen AW. Tumour vascularization: sprouting angiogenesis and beyond. Cancer Metastasis Rev (2007) 26:489–502. 10.1007/s10555-007-9094-7 17717633 PMC2797856

[70] Strasner A Karin M. Immune infiltration and prostate cancer. Front Oncol (2015) 5:128. 10.3389/fonc.2015.00128 26217583 PMC4495337

[71] Yang KQ Liu Y Huang QH Mo N Zhang QY Meng QG Bone marrow-derived mesenchymal stem cells induced by inflammatory cytokines produce angiogenetic factors and promote prostate cancer growth. BMC Cancer (2017) 17:878. 10.1186/s12885-017-3879-z 29268703 PMC5740893

[72] Zhang J Lu Y Pienta KJ. Multiple roles of chemokine (C-C motif) ligand 2 in promoting prostate cancer growth. J Natl Cancer Inst (2010) 102:522–8. 10.1093/jnci/djq044 20233997 PMC2857800

[73] Mizutani K Sud S McGregor NA Martinovski G Rice BT Craig MJ The chemokine CCL2 increases prostate tumor growth and bone metastasis through macrophage and osteoclast recruitment. Neoplasia (2009) 11:1235–42. 10.1593/neo.09988 19881959 PMC2767225

[74] Loberg RD Ying C Craig M Yan L Snyder LA Pienta KJ. CCL2 as an important mediator of prostate cancer growth in vivo through the regulation of macrophage infiltration. Neoplasia (2007) 9:556–62. 10.1593/neo.07307 17710158 PMC1939930

[75] Escamilla J Schokrpur S Liu C Priceman SJ Moughon D Jiang Z CSF1 receptor targeting in prostate cancer reverses macrophage-mediated resistance to androgen blockade therapy. Cancer Res (2015) 75:950–62. 10.1158/0008-5472.CAN-14-0992 25736687 PMC4359956

[76] Priceman SJ Sung JL Shaposhnik Z Burton JB Torres-Collado AX Moughon DL Targeting distinct tumor-infiltrating myeloid cells by inhibiting CSF-1 receptor: combating tumor evasion of antiangiogenic therapy. Blood (2010) 115:1461–71. 10.1182/blood-2009-08-237412 20008303 PMC2826767

[77] Xu J Escamilla J Mok S David J Priceman S West B CSF1R signaling blockade stanches tumor-infiltrating myeloid cells and improves the efficacy of radiotherapy in prostate cancer. Cancer Res (2013) 73:2782–94. 10.1158/0008-5472.CAN-12-3981 23418320 PMC4097014

[78] Cannarile MA Weisser M Jacob W Jegg AM Ries CH Ruttinger D. Colony-stimulating factor 1 receptor (CSF1R) inhibitors in cancer therapy. J Immunother Cancer (2017) 5:53. 10.1186/s40425-017-0257-y 28716061 PMC5514481

[79] Redente EF Dwyer-Nield LD Merrick DT Raina K Agarwal R Pao W Tumor progression stage and anatomical site regulate tumor-associated macrophage and bone marrow-derived monocyte polarization. Am J Pathol (2010) 176:2972–85. 10.2353/ajpath.2010.090879 20431028 PMC2877857

[80] Lin EY Nguyen AV Russell RG Pollard JW Colony-stimulating factor 1 promotes progression of mammary tumors to malignancy. J Exp Med (2001) 193:727–40. 10.1084/jem.193.6.727 11257139 PMC2193412

[81] Qian BZ Li J Zhang H Kitamura T Zhang J Campion LR CCL2 recruits inflammatory monocytes to facilitate breast-tumour metastasis. Nature (2011) 475:222–5. 10.1038/nature10138 21654748 PMC3208506

[82] Allavena P Sica A Solinas G Porta C Mantovani A. The inflammatory micro-environment in tumor progression: the role of tumor-associated macrophages. Crit Rev Oncol Hematol (2008) 66:1–9. 10.1016/j.critrevonc.2007.07.004 17913510

[83] Condeelis J Pollard JW. Macrophages: obligate partners for tumor cell migration, invasion, and metastasis. Cell (2006) 124:263–6. 10.1016/j.cell.2006.01.007 16439202

[84] Wan YY Flavell RA. TGF-beta and regulatory T cell in immunity and autoimmunity. J Clin Immunol (2008) 28:647–59. 10.1007/s10875-008-9251-y 18792765 PMC2837280

[85] Zhang L Yi H Xia XP Zhao Y. Transforming growth factor-beta: an important role in CD4+CD25+ regulatory T cells and immune tolerance. Autoimmunity (2006) 39:269–76. 10.1080/08916930600753903 16891215

[86] Wang X Lee SO Xia S Jiang Q Luo J Li L Endothelial cells enhance prostate cancer metastasis via IL-6–>androgen receptor–>TGF-beta–>MMP-9 signals. Mol Cancer Ther (2013) 12:1026–37. 10.1158/1535-7163.MCT-12-0895 23536722 PMC3782851

[87] Fiorio Pla A Brossa A Bernardini M Genova T Grolez G Villers A Differential sensitivity of prostate tumor derived endothelial cells to sorafenib and sunitinib. BMC Cancer (2014) 14:939. 10.1186/1471-2407-14-939 25494980 PMC4295225

[88] Shangguan L Ti X Krause U Hai B Zhao Y Yang Z Inhibition of TGF-beta/Smad signaling by BAMBI blocks differentiation of human mesenchymal stem cells to carcinoma-associated fibroblasts and abolishes their protumor effects. Stem Cells (2012) 30:2810–9. 10.1002/stem.1251 23034983

[89] Geiger R Rieckmann JC Wolf T Basso C Feng Y Fuhrer T L-arginine modulates T cell metabolism and enhances survival and anti-tumor activity. Cell (2016) 167:829–842.e13. 10.1016/j.cell.2016.09.031 27745970 PMC5075284

[90] Niedbala W Cai B Liew FY. Role of nitric oxide in the regulation of T cell functions. Ann Rheum Dis (2006) 65(Suppl 3):iii37–iii40. 10.1136/ard.2006.058446 17038470 PMC1798386

[91] Niedbala W Wei XQ Campbell C Thomson D Komai-Koma M Liew FY. Nitric oxide preferentially induces type 1 T cell differentiation by selectively up-regulating IL-12 receptor beta 2 expression via cGMP. Proc Natl Acad Sci U S A (2002) 99:16186–91. 10.1073/pnas.252464599 12451176 PMC138586

[92] Shiga K Hara M Nagasaki T Sato T Takahashi H Takeyama H Cancer-associated fibroblasts: their characteristics and their roles in tumor growth. Cancers (Basel) (2015) 7:2443–58. 10.3390/cancers7040902 26690480 PMC4695902

[93] Jung Y Kim JK Shiozawa Y Wang J Mishra A Joseph J Recruitment of mesenchymal stem cells into prostate tumours promotes metastasis. Nat Commun (2013) 4:1795. 10.1038/ncomms2766 23653207 PMC3649763

[94] Kaplan RN Psaila B Lyden D. Bone marrow cells in the “pre-metastatic niche”: within bone and beyond. Cancer Metastasis Rev (2006) 25:521–9. 10.1007/s10555-006-9036-9 17186383

[95] Zoni E van der Pluijm G. The role of microRNAs in bone metastasis. J Bone Oncol (2016) 5:104–8. 10.1016/j.jbo.2016.04.002 27761367 PMC5063223

[96] Weidle HU Birzele F Kollmorgen G RÜGer R. The multiple roles of exosomes in metastasis. Cancer Genomics Proteomics (2017) 14:1–16. 10.21873/cgp.20015 28031234 PMC5267497

[97] Sanchez CA Andahur EI Valenzuela R Castellon EA Fulla JA Ramos CG Exosomes from bulk and stem cells from human prostate cancer have a differential microRNA content that contributes cooperatively over local and pre-metastatic niche. Oncotarget (2016) 7:3993–4008. 10.18632/oncotarget.6540 26675257 PMC4826185

[98] Rauschenberger L Staar D Thom K Scharf C Venz S Homuth G Exosomal particles secreted by prostate cancer cells are potent mRNA and protein vehicles for the interference of tumor and tumor environment. Prostate (2016) 76:409–24. 10.1002/pros.23132 26643154

[99] Mathivanan S Fahner CJ Reid GE Simpson RJ ExoCarta 2012: database of exosomal proteins, RNA and lipids. Nucleic Acids Res (2012) 40:D1241–4. 10.1093/nar/gkr828 21989406 PMC3245025

[100] Pan J Ding M Xu K Yang C Mao LJ. Exosomes in diagnosis and therapy of prostate cancer. Oncotarget (2017) 8:97693–700. 10.18632/oncotarget.18532 29228644 PMC5722596

[101] Karlsson T Lundholm M Widmark A Persson E. Tumor cell-derived exosomes from the prostate cancer cell line TRAMP-C1 impair osteoclast formation and differentiation. PLoS One (2016) 11:e0166284. 10.1371/journal.pone.0166284 27832183 PMC5104397

[102] Soki FN Koh AJ Jones JD Kim YW Dai J Keller ET Polarization of prostate cancer-associated macrophages is induced by milk fat globule-EGF factor 8 (MFG-E8)-mediated efferocytosis. J Biol Chem (2014) 289:24560–72. 10.1074/jbc.M114.571620 25006249 PMC4148880

[103] Aziz M Jacob A Matsuda A Wu R Zhou M Dong W Pre-treatment of recombinant mouse MFG-E8 downregulates LPS-induced TNF-alpha production in macrophages via STAT3-mediated SOCS3 activation. PLoS One (2011) 6:e27685 10.1371/journal.pone.0027685 22114683 PMC3217009

[104] Bonci D Coppola V Patrizii M Addario A Cannistraci A Francescangeli F A microRNA code for prostate cancer metastasis. Oncogene (2016) 35:1180–92. 10.1038/onc.2015.176 26073083 PMC4803473

[105] Sugatani T Vacher J Hruska KA. A microRNA expression signature of osteoclastogenesis. Blood (2011) 117:3648–57. 10.1182/blood-2010-10-311415 21273303 PMC3072882

[106] Shiozawa Y Eber MR Berry JE Taichman RS. Bone marrow as a metastatic niche for disseminated tumor cells from solid tumors. Bonekey Rep (2015) 4:689. 10.1038/bonekey.2015.57 26029360 PMC4440229

[107] Sousa S Määttä J The role of tumour-associated macrophages in bone metastasis. J Bone Oncol (2016) 5:135–8. 10.1016/j.jbo.2016.03.004 27761375 PMC5063225

[108] Qian B-Z Pollard JW. Macrophage diversity enhances tumor progression and metastasis. Cell (2010) 141:39–51. 10.1016/j.cell.2010.03.014 20371344 PMC4994190

[109] Zarif JC Taichman RS Pienta KJ. TAM macrophages promote growth and metastasis within the cancer ecosystem. Oncoimmunology (2014) 3:e941734. 10.4161/21624011.2014.941734 25954596 PMC4341447

[110] Teti A. Mechanisms of osteoclast-dependent bone formation. Bonekey Rep (2013) 2:449. 10.1038/bonekey.2013.183 24422142 PMC3872977

[111] Mundy GR The effects of TGF-beta on bone. Ciba Found Symp (1991) 157:137–43; discussion 143–51. 2070682

[112] Keller ET Brown J. Prostate cancer bone metastases promote both osteolytic and osteoblastic activity. J Cell Biochem (2004) 91:718–29. 10.1002/jcb.10662 14991763

[113] Zhang Z Jimi E Bothwell AL. Receptor activator of NF-kappa B ligand stimulates recruitment of SHP-1 to the complex containing TNFR-associated factor 6 that regulates osteoclastogenesis. J Immunol (2003) 171:3620–6. 10.4049/jimmunol.171.7.3620 14500659

[114] Takeshita S Kaji K Kudo A. Identification and characterization of the new osteoclast progenitor with macrophage phenotypes being able to differentiate into mature osteoclasts. J Bone Miner Res (2000) 15:1477–88. 10.1359/jbmr.2000.15.8.1477 10934646

[115] Wu AC He Y Broomfield A Paatan NJ Harrington BS Tseng H-W CD169+ macrophages mediate pathological formation of woven bone in skeletal lesions of prostate cancer. J Pathol (2016) 239:218–30. 10.1002/path.4718 27174786

[116] Soki FN Cho SW Kim YW Jones JD Park SI Koh AJ Bone marrow macrophages support prostate cancer growth in bone. Oncotarget (2015) 6:35782–96. 10.18632/oncotarget.6042 26459393 PMC4742141

[117] Yang L Zhang Y. Tumor-associated macrophages: from basic research to clinical application. J Hematol Oncol (2017) 10:58. 10.1186/s13045-017-0430-2 28241846 PMC5329931

[118] Antonarakis ES Lu C Wang H Luber B Nakazawa M Roeser JC AR-V7 and resistance to enzalutamide and abiraterone in prostate cancer. N Engl J Med (2014) 371:1028–38. 10.1056/NEJMoa1315815 25184630 PMC4201502

[119] Hoefer J Akbor M Handle F Ofer P Puhr M Parson W Critical role of androgen receptor level in prostate cancer cell resistance to new generation antiandrogen enzalutamide. Oncotarget (2016) 7:59781–94. 10.18632/oncotarget.10926 27486973 PMC5312348

[120] Seitz AK Thoene S Bietenbeck A Nawroth R Tauber R Thalgott M AR-V7 in peripheral whole blood of patients with castration-resistant prostate cancer: association with treatment-specific outcome under abiraterone and enzalutamide. Eur Urol (2017) 72:828–34. 10.1016/j.eururo.2017.07.024 28818355

[121] Lin TH Izumi K Lee SO Lin WJ Yeh S Chang C. Anti-androgen receptor ASC-J9 versus anti-androgens MDV3100 (enzalutamide) or casodex (bicalutamide) leads to opposite effects on prostate cancer metastasis via differential modulation of macrophage infiltration and STAT3-CCL2 signaling. Cell Death Dis (2013) 4:e764. 10.1038/cddis.2013.270 23928703 PMC3763432

[122] Lin TH Lee SO Niu Y Xu D Liang L Li L Differential androgen deprivation therapies with anti-androgens casodex/bicalutamide or MDV3100/enzalutamide versus anti-androgen receptor ASC-J9(R) lead to promotion versus suppression of prostate cancer metastasis. J Biol Chem (2013) 288:19359–69. 10.1074/jbc.M113.477216 23687298 PMC3707641

[123] Wang XJ Zhuo J Luo GH Zhu YP Yu DJ Zhao RZ Androgen deprivation accelerates the prostatic urethra wound healing after thulium laser resection of the prostate by promoting re-epithelialization and regulating the macrophage polarization. Prostate (2017) 77:708–17. 10.1002/pros.23301 28168722

[124] Wang A Karunasinghe N Plank L Zhu S Osborne S Bishop K Effect of androgen deprivation therapy on bone mineral density in a prostate cancer cohort in New Zealand: a pilot study. Clin Med Insights Oncol (2017) 11:1179554917733449. 10.1177/1179554917733449 29051709 PMC5638161

[125] Crawford ED Higano CS Shore ND Hussain M Petrylak DP. Treating patients with metastatic castration resistant prostate cancer: a comprehensive review of available therapies. J Urol (2015) 194:1537–47. 10.1016/j.juro.2015.06.106 26196735

[126] Paller CJ Antonarakis ES. Cabazitaxel: a novel second-line treatment for metastatic castration-resistant prostate cancer. Drug Des Devel Ther (2011) 5:117–24. 10.2147/DDDT.S13029 21448449 PMC3063116

[127] Colloca G Venturino A Checcaglini F. Second-line chemotherapy in metastatic docetaxel-resistant prostate cancer: a review. Med Oncol (2012) 29:776–85. 10.1007/s12032-011-9855-6 21336988

[128] Petrylak DP. Practical guide to the use of chemotherapy in castration resistant prostate cancer. Can J Urol (2014) 21:77–83. 24775728

[129] Yin Y Huang X Lynn KD Thorpe PE. Phosphatidylserine-targeting antibody induces M1 macrophage polarization and promotes myeloid-derived suppressor cell differentiation. Cancer Immunol Res (2013) 1:256–68. 10.1158/2326-6066.CIR-13-0073 24777853

[130] Kodumudi KN Woan K Gilvary DL Sahakian E Wei S Djeu JY. A novel chemoimmunomodulating property of docetaxel: suppression of myeloid-derived suppressor cells in tumor bearers. Clin Cancer Res (2010) 16:4583–94. 10.1158/1078-0432.CCR-10-0733 20702612 PMC3874864

[131] Mahon KL Lin HM Castillo L Lee BY Lee-Ng M Chatfield MD Cytokine profiling of docetaxel-resistant castration-resistant prostate cancer. Br J Cancer (2015) 112:1340–8. 10.1038/bjc.2015.74 25867259 PMC4402456

[132] Wolff B Burns AR Middleton J Rot A Endothelial cell "memory" of inflammatory stimulation: human venular endothelial cells store interleukin 8 in Weibel-Palade bodies. J Exp Med (1998) 188:1757–62. 10.1084/jem.188.9.1757 9802987 PMC2212526

[133] Utgaard JO Jahnsen FL Bakka A Brandtzaeg P Haraldsen G. Rapid secretion of prestored interleukin 8 from Weibel-Palade bodies of microvascular endothelial cells. J Exp Med (1998) 188:1751–6. 10.1084/jem.188.9.1751 9802986 PMC2212514

[134] Brat DJ Bellail AC Van Meir EG. The role of interleukin-8 and its receptors in gliomagenesis and tumoral angiogenesis. Neuro Oncol (2005) 7:122–33. 10.1215/S1152851704001061 15831231 PMC1871893

[135] Magadoux L Isambert N Plenchette S Jeannin JF Laurens V. Emerging targets to monitor and overcome docetaxel resistance in castration resistant prostate cancer (review). Int J Oncol (2014) 45:919–28. 10.3892/ijo.2014.2517 24969394

[136] Lu Y Cai Z Galson DL Xiao G Liu Y George DE Monocyte chemotactic protein-1 (MCP-1) acts as a paracrine and autocrine factor for prostate cancer growth and invasion. Prostate (2006) 66:1311–8. 10.1002/pros.20464 16705739

[137] Roca H Varsos ZS Pienta KJ. CCL2 is a negative regulator of AMP-activated protein kinase to sustain mTOR complex-1 activation, survivin expression, and cell survival in human prostate cancer PC3 cells. Neoplasia (2009) 11:1309–17. 10.1593/neo.09936 20019839 PMC2794512

[138] Loberg RD Day LL Harwood J Ying C St John LN Giles R CCL2 is a potent regulator of prostate cancer cell migration and proliferation. Neoplasia (2006) 8:578–86. 10.1593/neo.06280 16867220 PMC1601934

[139] Qian DZ Rademacher BL Pittsenbarger J Huang CY Myrthue A Higano CS CCL2 is induced by chemotherapy and protects prostate cancer cells from docetaxel-induced cytotoxicity. Prostate (2010) 70:433–42. 10.1002/pros.21077 19866475 PMC2931415

[140] Takahashi M Mizoguchi T Uehara S Nakamichi Y Yang S Naramoto H Docetaxel inhibits bone resorption through suppression of osteoclast formation and function in different manners. J Bone Miner Metab (2009) 27:24–35. 10.1007/s00774-008-0013-y 19082914

[141] Yeku O Slovin SF Radium-223 and concomitant therapies: prospects and prudence. Transl Androl Urol (2016) 5:968–70. 10.21037/tau.2016.11.04 28078234 PMC5182218

[142] Wenter V Herlemann A Fendler WP Ilhan H Tirichter N Bartenstein P Radium-223 for primary bone metastases in patients with hormone-sensitive prostate cancer after radical prostatectomy. Oncotarget (2017) 8:44131–40. 10.18632/oncotarget.17311 28484088 PMC5546468

[143] Parker CC Coleman RE Sartor O Vogelzang NJ Bottomley D Heinrich D Three-year safety of radium-223 dichloride in patients with castration-resistant prostate cancer and symptomatic bone metastases from phase 3 randomized alpharadin in Symptomatic Prostate Cancer Trial. Eur Urol (2018) 73:427–35. 10.1016/j.eururo.2017.06.021 28705540

[144] Armstrong CW Maxwell PJ Ong CW Redmond KM McCann C Neisen J PTEN deficiency promotes macrophage infiltration and hypersensitivity of prostate cancer to IAP antagonist/radiation combination therapy. Oncotarget (2016) 7:7885–98. 10.18632/oncotarget.6955 26799286 PMC4884961

[145] Teresa Pinto A Laranjeiro Pinto M Patricia Cardoso A Monteiro C Teixeira Pinto M Filipe Maia A Ionizing radiation modulates human macrophages towards a pro-inflammatory phenotype preserving their pro-invasive and pro-angiogenic capacities. Sci Rep (2016) 6:18765. 10.1038/srep18765 26735768 PMC4702523

[146] Vanpouille-Box C Diamond JM Pilones KA Zavadil J Babb JS Formenti SC TGFbeta is a master regulator of radiation therapy-induced antitumor immunity. Cancer Res (2015) 75:2232–42. 10.1158/0008-5472.CAN-14-3511 25858148 PMC4522159

[147] Mizokami A Gotoh A Yamada H Keller ET Matsumoto T. Tumor necrosis factor-alpha represses androgen sensitivity in the LNCaP prostate cancer cell line. J Urol (2000) 164:800–5. 10.1016/S0022-5347(05)67318-1 10953159

[148] Michalaki V Syrigos K Charles P Waxman J. Serum levels of IL-6 and TNF-alpha correlate with clinicopathological features and patient survival in patients with prostate cancer. Br J Cancer (2004) 90:2312–6. 10.1038/sj.bjc.6601814 15150588 PMC2409519

[149] Sethi G Sung B Aggarwal BB TNF: a master switch for inflammation to cancer. Front Biosci (2008) 13:5094–107. 10.2741/3066 18508572

[150] Lam J Takeshita S Barker JE Kanagawa O Ross FP Teitelbaum SL. TNF-alpha induces osteoclastogenesis by direct stimulation of macrophages exposed to permissive levels of RANK ligand. J Clin Invest (2000) 106:1481–8. 10.1172/JCI11176 11120755 PMC387259

[151] Kobayashi K Takahashi N Jimi E Udagawa N Takami M Kotake S Tumor necrosis factor alpha stimulates osteoclast differentiation by a mechanism independent of the ODF/RANKL-RANK interaction. J Exp Med (2000) 191:275–86. 10.1084/jem.191.2.275 10637272 PMC2195746

[152] Zhang YH Heulsmann A Tondravi MM Mukherjee A Abu-Amer Y. Tumor necrosis factor-alpha (TNF) stimulates RANKL-induced osteoclastogenesis via coupling of TNF type 1 receptor and RANK signaling pathways. J Biol Chem (2001) 276:563–8. 10.1074/jbc.M008198200 11032840

[153] Osta B Benedetti G Miossec P Classical and paradoxical effects of TNF-alpha on bone homeostasis. Front Immunol (2014) 5:48 10.3389/fimmu.2014.00048 24592264 PMC3923157

[154] Joseph IB Isaacs JT. Macrophage role in the anti-prostate cancer response to one class of antiangiogenic agents. J Natl Cancer Inst (1998) 90:1648–53. 10.1093/jnci/90.21.1648 9811314

[155] Zhang J Patel L Pienta KJ. CC chemokine ligand 2 (CCL2) promotes prostate cancer tumorigenesis and metastasis. Cytokine Growth Factor Rev (2010) 21:41–8. 10.1016/j.cytogfr.2009.11.009 20005149 PMC2857769

[156] Craig MJ Loberg RD. CCL2 (monocyte chemoattractant protein-1) in cancer bone metastases. Cancer Metastasis Rev (2006) 25:611–9. 10.1007/s10555-006-9027-x 17160712

[157] Li X Loberg R Liao J Ying C Snyder LA Pienta KJ A destructive cascade mediated by CCL2 facilitates prostate cancer growth in bone. Cancer Res (2009) 69:1685–92. 10.1158/0008-5472.CAN-08-2164 19176388 PMC2698812

[158] Loberg RD Ying C Craig M Day LL Sargent E Neeley C Targeting CCL2 with systemic delivery of neutralizing antibodies induces prostate cancer tumor regression in vivo. Cancer Res (2007) 67:9417–24. 10.1158/0008-5472.CAN-07-1286 17909051

[159] Sandhu SK Papadopoulos K Fong PC Patnaik A Messiou C Olmos D A first-in-human, first-in-class, phase I study of carlumab (CNTO 888), a human monoclonal antibody against CC-chemokine ligand 2 in patients with solid tumors. Cancer Chemother Pharmacol (2013) 71:1041–50. 10.1007/s00280-013-2099-8 23385782

[160] Zhang J Patel L Pienta KJ. Targeting chemokine (C-C motif) ligand 2 (CCL2) as an example of translation of cancer molecular biology to the clinic. Prog Mol Biol Transl Sci (2010) 95:31–53. 10.1016/B978-0-12-385071-3.00003-4 21075328 PMC3197817

[161] Pienta KJ Machiels JP Schrijvers D Alekseev B Shkolnik M Crabb SJ Phase 2 study of carlumab (CNTO 888), a human monoclonal antibody against CC-chemokine ligand 2 (CCL2), in metastatic castration-resistant prostate cancer. Invest New Drugs (2013) 31:760–8. 10.1007/s10637-012-9869-8 22907596

[162] Kirk PS Koreckij T Nguyen HM Brown LG Snyder LA Vessella RL Inhibition of CCL2 signaling in combination with docetaxel treatment has profound inhibitory effects on prostate cancer growth in bone. Int J Mol Sci (2013) 14:10483–96. 10.3390/ijms140510483 23698775 PMC3676850

[163] Rozel S Galban CJ Nicolay K Lee KC Sud S Neeley C Synergy between anti-CCL2 and docetaxel as determined by DW-MRI in a metastatic bone cancer model. J Cell Biochem (2009) 107:58–64. 10.1002/jcb.22056 19259948 PMC4293017

[164] Brana I Calles A LoRusso PM Yee LK Puchalski TA Seetharam S Carlumab, an anti-C-C chemokine ligand 2 monoclonal antibody, in combination with four chemotherapy regimens for the treatment of patients with solid tumors: an open-label, multicenter phase 1b study. Target Oncol (2015) 10:111–23. 10.1007/s11523-014-0320-2 24928772

[165] Li X Qin L Bergenstock M Bevelock LM Novack DV Partridge NC. Parathyroid hormone stimulates osteoblastic expression of MCP-1 to recruit and increase the fusion of pre/osteoclasts. J Biol Chem (2007) 282:33098–106. 10.1074/jbc.M611781200 17690108

[166] Craig M Ying C Loberg RD. Co-inoculation of prostate cancer cells with U937 enhances tumor growth and angiogenesis in vivo. J Cell Biochem (2008) 103:1–8. 10.1002/jcb.21379 17541941

[167] DeNardo DG Barreto JB Andreu P Vasquez L Tawfik D Kolhatkar N CD4(+) T cells regulate pulmonary metastasis of mammary carcinomas by enhancing protumor properties of macrophages. Cancer Cell (2009) 16:91–102. 10.1016/j.ccr.2009.06.018 19647220 PMC2778576

[168] Apte SH Groves P Olver S Baz A Doolan DL Kelso A IFN-gamma inhibits IL-4-induced type 2 cytokine expression by CD8 T cells in vivo and modulates the anti-tumor response. J Immunol (2010) 185:998–1004. 10.4049/jimmunol.0903372 20562261

[169] Goldstein R Hanley C Morris J Cahill D Chandra A Harper P Clinical investigation of the role of interleukin-4 and interleukin-13 in the evolution of prostate cancer. Cancers (Basel) (2011) 3:4281–93. 10.3390/cancers3044281 24213139 PMC3763424

[170] Toi M Bicknell R Harris AL. Inhibition of colon and breast carcinoma cell growth by interleukin-4. Cancer Res (1992) 52:275–9. 1728401

[171] Atkins MB Vachino G Tilg HJ Karp DD Robert NJ Kappler K Phase I evaluation of thrice-daily intravenous bolus interleukin-4 in patients with refractory malignancy. J Clin Oncol (1992) 10:1802–9. 10.1200/JCO.1992.10.11.1802 1403061

[172] Takeshi U Sadar MD Suzuki H Akakura K Sakamoto S Shimbo M Interleukin-4 in patients with prostate cancer. Anticancer Res (2005) 25:4595–8. 16334148

[173] Lee SO Pinder E Chun JY Lou W Sun M Gao AC. Interleukin-4 stimulates androgen-independent growth in LNCaP human prostate cancer cells. Prostate (2008) 68:85–91. 10.1002/pros.20691 18008330

[174] Frost A Jonsson KB Brandstrom H Ljunghall S Nilsson O Ljunggren O. Interleukin (IL)-13 and IL-4 inhibit proliferation and stimulate IL-6 formation in human osteoblasts: evidence for involvement of receptor subunits IL-13R, IL-13Ralpha, and IL-4Ralpha. Bone (2001) 28:268–74. 10.1016/S8756-3282(00)00449-X 11248656

[175] Yamada A Takami M Kawawa T Yasuhara R Zhao B Mochizuki A Interleukin-4 inhibition of osteoclast differentiation is stronger than that of interleukin-13 and they are equivalent for induction of osteoprotegerin production from osteoblasts. Immunology (2007) 120:573–9. 10.1111/j.1365-2567.2006.02538.x 17343616 PMC2265899

[176] Shree T Olson OC Elie BT Kester JC Garfall AL Simpson K Macrophages and cathepsin proteases blunt chemotherapeutic response in breast cancer. Genes Dev (2011) 25:2465–79. 10.1101/gad.180331.111 22156207 PMC3243057

[177] DeNardo DG Brennan DJ Rexhepaj E Ruffell B Shiao SL Madden SF Leukocyte complexity predicts breast cancer survival and functionally regulates response to chemotherapy. Cancer Discov (2011) 1:54–67. 10.1158/2159-8274.CD-10-0028 22039576 PMC3203524

[178] Brahmi M Vinceneux A Cassier PA. Current systemic treatment options for tenosynovial giant cell tumor/pigmented villonodular synovitis: targeting the CSF1/CSF1R axis. Curr Treat Options Oncol (2016) 17:10. 10.1007/s11864-015-0385-x 26820289

[179] Tap WD Wainberg ZA Anthony SP Ibrahim PN Zhang C Healey JH Structure-guided blockade of CSF1R kinase in tenosynovial giant-cell tumor. N Engl J Med (2015) 373:428–37. 10.1056/NEJMoa1411366 26222558

[180] Sauter KA Pridans C Sehgal A Tsai YT Bradford BM Raza S Pleiotropic effects of extended blockade of CSF1R signaling in adult mice. J Leukoc Biol (2014) 96:265–74. 10.1189/jlb.2A0114-006R 24652541 PMC4378363

